# Uncovering further diversity of *Ochoterenatrema* Caballero, 1943 (Digenea: Lecithodendriidae) in South American bats

**DOI:** 10.1007/s11230-024-10165-0

**Published:** 2024-05-28

**Authors:** Vasyl V. Tkach, Roxanne Gasperetti, Thayane F. Fernandes, Carlos A. Carrión-Bonilla, Joseph A. Cook, Tyler J. Achatz

**Affiliations:** 1 Department of Biology, University of North Dakota, 10 Cornell Street, Grand Forks, ND 58202 USA; 2 Department of Biology and Museum of Natural History, University of Wisconsin-Stevens Point, Stevens Point, WI 54481 USA; 3 Faculdade Facimp Wyden, Imperatriz, Maranhão 65914-335 Brazil; 4 Museo de Zoología QCAZ, Facultad de Ciencias Biológicas, Pontificia Universidad Católica del Ecuador, Quito, Ecuador; 5 Museum of Southwestern Biology and Department of Biology, University of New Mexico, Albuquerque, NM 87131 USA; 6 Department of Natural Sciences, Middle Georgia State University, Macon, GA 31206 USA

## Abstract

*Ochoterenatrema* Caballero, 1943 is a genus of lecithodendriid digeneans that prior to this study included 8 species parasitic in bats in the Western Hemisphere. Species of *Ochoterenatrema* possess a unique morphological feature in form of the pseudogonotyl on the sinistral side of the ventral sucker. In this study, we describe 2 new species of *Ochoterenatrema* from bats in Ecuador. The new species are readily differentiated from their congeners by a combination of morphological characters, including the distribution of vitelline follicles, length of oesophagus, sucker ratio and the body shape, among other features. We have generated partial nuclear 28S rDNA and mitochondrial *cox*1 gene DNA sequences from both new species. The newly obtained sequences were used to differentiate among species and study the phylogenetic interrelationships among *Ochoterenatrema* spp. The internal topology of the clade was weakly supported, although the *cox*1 tree was much better resolved than the 28S tree. Comparison of sequences revealed 0–1.2% interspecific divergence in 28S and 3.3–20.5% interspecific divergence in *cox*1 among *Ochoterenatrema* spp. The new findings demonstrate that bats in South America likely harbor multiple additional undescribed species of *Ochoterenatrema*. More extensive sampling from broader geographic and host ranges, especially in North America, should allow for a better understanding of the evolution of host associations and morphological traits of this lineage of lecithodendriid digeneans.

## Introduction

*Ochoterenatrema* Caballero, 1943 is a relatively small genus of digeneans belonging to the highly diverse family Lecithodendriidae Lühe, 1901. Members of *Ochoterenatrema* are parasitic exclusively in bats in the Western Hemisphere. They are characterized by the presence of a unique morphological feature among lecithodendriids, namely the pseudogonotyl which is formed by thickened tegument on the sinistral side of the ventral sucker (Lotz & Font, 2008). Some authors have mistakenly referred to this structure as the genital pore and its function remains unknown (see references in Fernandes et al., 2022). Until recently, molecular data on *Ochoterenatrema* were lacking and its phylogenetic affinities were unknown. Fernandes et al. (2022) provided the first DNA sequence data for 4 species of *Ochoterenatrema* from South and North America. Their phylogenetic analysis confirmed the monophyly of *Ochoterenatrema* and demonstrated its position as the closest taxon to the genus *Lecithodendrium*. In addition, Fernandes et al. (2022) described 2 new species, *Ochoterenatrema sphaerula* Fernandes, Melo, Santos, Achatz et Tkach, 2022 and *Ochoterenatrema gracilis* Fernandes, Achatz, McAllister et Tkach, 2022, and provided quality morphological descriptions and illustrations of *Ochoterenatrema diminutum* (Chandler, 1938), *Ochoterenatrema fraternum* Freitas et Ibañez, 1963 and *Ochoterenatrema* cf. *labda* Caballero, 1943.

Eight species of *Ochoterenatrema* have been described, 3 of them from the Nearctic and 5 from the Neotropics (original descriptions in Macy, 1936, 1938; Chandler, 1938; Caballero, 1943; Freitas, 1957; Freitas & Ibañez, 1963; Fernandes et al., 2022). Fernandes et al. (2022) hypothesized that *Ochoterenatrema* spp. may be more diverse in Neotropics than currently known. In the course of parasitological examination of bats in Ecuador, we discovered 2 previously unknown species of *Ochoterenatrema*. Herein, we provide morphological descriptions of the new species accompanied by molecular differentiation and phylogenetic analysis based on partial sequences of the nuclear large ribosomal subunit (28S) gene and the mitochondrial cytochrome *c* oxidase 1 (*cox*1) DNA gene.

## Materials and methods

*Morphological data*

Specimens of 2 new species of *Ochoterenatrema* were collected from the intestines of bats in Yasuni National Park, Orellana Province and the Centro Científico Rio Palenque, Los Ríos Province, and Luis Vargas Torres, community of Playa de Oro, Esmeraldas province, Ecuador in 2016 and 2017. Bats were trapped using mist nets. Digeneans were collected live, rinsed in saline, heat-killed with hot water, and immediately fixed in 70% ethanol for morphological and molecular studies. Specimens for light microscopy were stained with aqueous alum carmine, dehydrated in an ethanol series of ascending concentrations, cleared in clove oil and mounted permanently in Damar gum (Lutz et al., 2017). The identification, measurements, and drawings of the specimens were made using an Olympus BX51 microscope (Olympus Corporation, Tokyo, Japan) equipped with DIC and digital imaging system. Drawings were made with the aid of a drawing tube. All measurements are in micrometers; forebody was measured as distance from anterior end of the body to the center of ventral sucker. The CV is a percentage value of the ratio of the standard deviation to the mean of a particular metric character. Characters with lower CV have values that are more stable around the mean than those with higher CV. The type specimens are deposited in the collection of the Museo de Zoología QCAZ, Facultad de Ciencias Biológicas, Pontificia Universidad Católica del Ecuador (QCAZI), Quito, Ecuador. Museum accession numbers are provided in the descriptions below.

*Molecular data*

Genomic DNA was extracted according to the protocol described by Tkach and Pawlowski (1999). An approximately 1,300 bp long fragment at the 5′ end of 28S was amplified by polymerase chain reactions (PCR) on a T100™ thermal cycler (Bio-Rad, Hercules, California, USA) using forward primer digL2 (5′–AAG CAT ATC ACT AAG CGG – 3′) and the reverse primer 1500R (5′–GCT ATC CTG AGG GAA ACT TCG–3′) (Tkach et al., 2003). A 396 bp long (upon trimming of primers) fragment of the *cox*1 gene was amplified using forward primer JB3 (5′–TTT GGG CAT CCT GAG GTT TAT–3′) and reverse primer JB4.5 (5` – TAA AGA AAG AAC ATA ATG AAA ATG – 3`) (Bowles et al., 1992). The PCRs were performed in a total volume of 25 µl using One-Taq quick load PCR mix from New England Biolabs (Ipswich, Massachusetts, USA) according to the manufacturer’s protocol and using an annealing temperature of 53°C for 28S and 45°C for *cox*1.

The PCR products were purified using Illustra ExoProStar PCR clean-up enzymatic kit (Cytiva, Marlborough, Massachusetts, USA) following the manufacturer's protocol. PCR products were cycle‐sequenced directly using BrightDye Terminator Cycle Sequencing Kit (MCLAB, South San Francisco, California, USA), Big Dye Sequencing Clean Up Kit (MCLAB) and run on an ABI Prism 3130™ automated capillary sequencer (Thermo Fisher Scientific, Waltham, Massachusetts, USA). The PCR primers and an additional internal forward primer 300F (5′–CAA GTA CCG TGA GGG AAA GTT G–3′) were used in sequencing reactions (Tkach et al., 2003) for the 28S fragment; PCR primers were used for sequencing for the *cox*1 fragment. Contiguous sequences were assembled using Sequencher ver. 4.2 (GeneCodes Corp., Ann Arbor, Michigan, USA) and submitted to GenBank (see Table 1 for accession numbers).

**Table 1:** Digenean species used in this study including their host species, geographical origin of material, and GenBank accession numbers.

Digenean species	Host species	Geographic origin	GenBank No.		Source
			28S	*cox*1	
*O. piriforme* n. sp.*	*Myotis diminutus*	Esmeraldos Province, Ecuador		PP069736, PP534964	Present study Present study
	*Myotis diminutus*	Los Ríos Province, Ecuador	PP069556	PP069737	Present study
*O. giovannionorei* n. sp.*	*Molossus molossus*	Yasuni National Park, Orellana Province, Ecuador	PP534962	PP534965	Present study
*O. gracilis*	*Perimyotis subflavus*	Oklahoma, USA	OM574910	OM641988	Fernandes et al. (2022)
*O. diminutum*	*Molossus molossus*	Pará State, Brazil	OM574911 OM574912	OM641989	Fernandes et al. (2022)
*O.* cf.* labda*	*Myotis* sp.	Mato Grosso State, Brazil	OM574913	OM641990	Fernandes et al. (2022)
*O. fraternum*	*Myotis diminutus*	Pichincha Province, Ecuador	OM574914	OM641991	Fernandes et al. (2022)
	*Myotis diminutus*	Santo Domingo de los Tsachilas Province, Ecuador	OM574915	OM641992	Fernandes et al. (2022)
	*Myotis diminutus*	Esmeraldos Province, Ecuador	PP534961	PP534963	Present study
*Cryptotropa acanthosauri*	*Acanthosaura lepidogaster*	Vietnam	OQ534009	OQ469314	Tkach et al. (2023)

Since the monophyly of the *Ochoterenatrema* has been confirmed in a recent publication by Fernandes et al. (2022), we limited our phylogenetic analyses to the interrelationships within the genus. Two phylogenetic analyses using 28S and *cox*1 sequences were performed. Newly obtained and previously published sequences were aligned using ClustalW implemented in MEGA7 (Kumar et al., 2016); both alignments were trimmed to the length of the shortest sequence. *Cryptotropa acanthosauri* Tkach, Chermak, Patitucci et Binh, 2023 was used as outgroup in both phylogenies based on the previously published phylogenies (Fernandes et al., 2022; Tkach et al., 2023). It was the closest species for which matching sequences were available for both 28S and *cox*1 regions; both sequences were obtained from the same isolate of *C. acanthosauri*.

The phylogenetic analyses were conducted using Bayesian inference (BI) as implemented in the MrBayes software ver. 3.2.6 (Ronquist & Huelsenbeck, 2003). The general time-reversible model with estimates of invariant sites and gamma-distributed among‐site variation (GTR + I + G) was identified as the best‐fitting nucleotide substitution model for both alignments using JMODELTEST 2 software (Darriba et al., 2012). The BI analyses were performed using MrBayes software as follows: Markov chain Monte Carlo (MCMC) chains were run for 3,000,000 generations with sample frequency set at 1,000. Log‐likelihood scores were plotted and only the final 75% of trees were used to produce the consensus trees by setting the “burn‐in” parameter at 750. This number of generations was considered sufficient because the standard deviation stabilized below 0.01. For convenience, we present posterior probabilities as percentages.

Pairwise sequence comparisons of *Ochoterenatrema* spp. were done using MEGA7.

## Results

*Species descriptions*

***Ochoterenatrema piriforme*** **n. sp.**

(Fig. 1A–C)

**Fig. 1:**
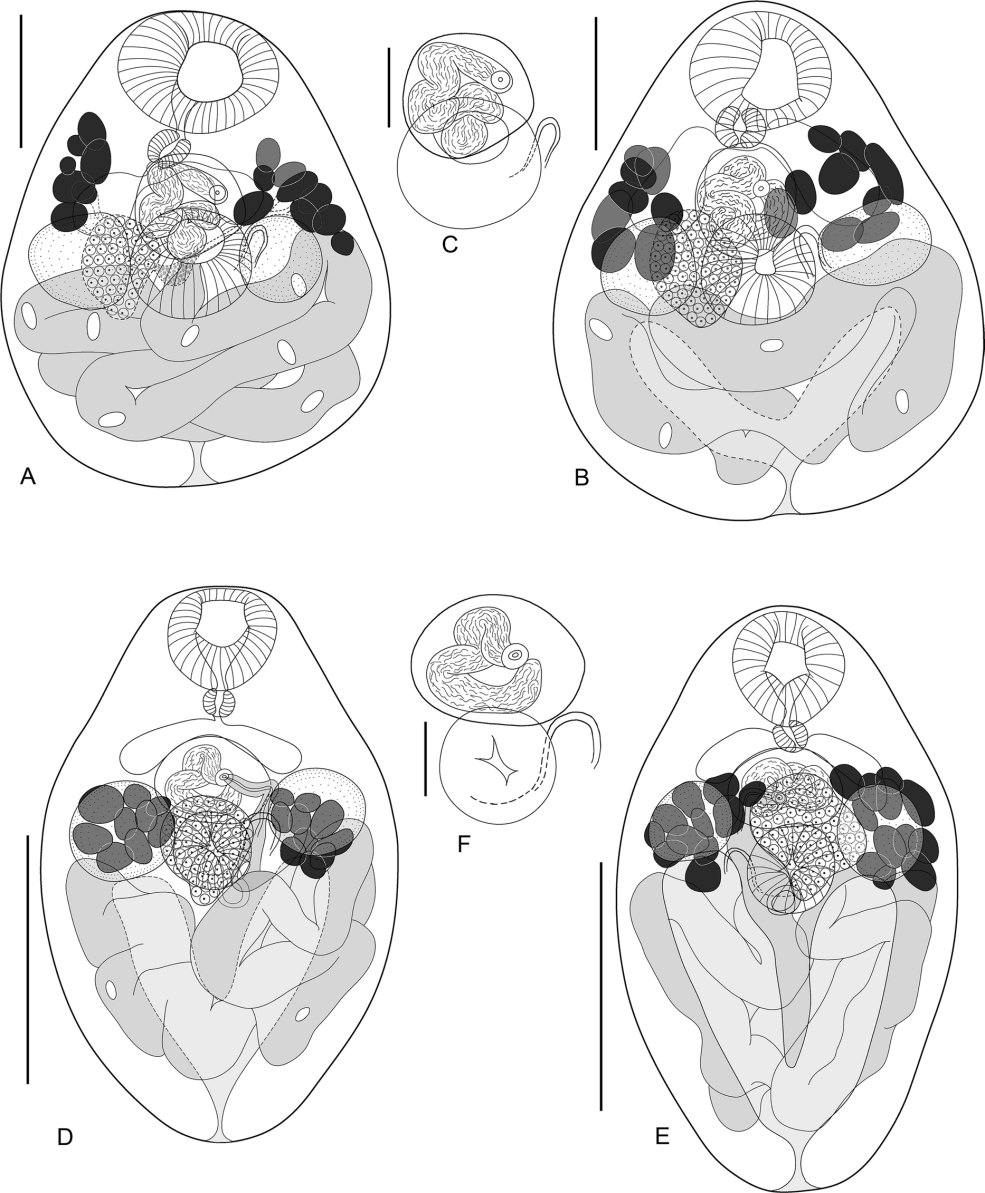
Morphology of *Ochoterenatrema piriforme* **n. sp.** and *Ochoterenatrema giovannionorei* **n. sp.** A, *O. piriforme* **n. sp.**, holotype, ventral view; B, *O. piriforme* **n. sp.**, paratype; C, *O. piriforme* **n. sp.**, pseudogonotyl, ventral view; D, *O. giovannionorei* **n. sp.**, holotype, ventral view; E, *O. giovannionorei* **n. sp.**, paratype, dorsal view; F, *O. giovannionorei* **n. sp.**, pseudogonotyl, ventral view. Scale bars: A, B = 100 µm, C, F = 50 µm, D, E = 500 µm.

*Type-host*: *Myotis diminutus* Moratelli et Wilson (Chiroptera Blumenbach, Vespertilionidae Gray).

*Type-locality:* Luis Vargas Torres, community of Playa de Oro, Esmeraldas Province, Ecuador (0°52′33″N, 78°47′41″W).

*Other localities*: Centro Científico Río Palenque, Los Ríos Province, Ecuador (0°35′17″S, 79°21′47″W).

*Type-material*: The type series consists of 14 fully mature specimens. Holotype: QCAZI 278977, small intestine, Luis Vargas Torres, community of Playa de Oro, Esmeraldas Province, Ecuador, 25 August 2017, coll. C. Carrión-Bonilla; paratypes: QCAZI 278978-278990, labels identical to the label of holotype.

*Site in host*: Small intestine.

*Representative DNA sequences in GenBank*: PP069556 (28S), PP069736–PP069737, PP534964 (*cox*1).

*ZooBank registration:* urn:lsid:zoobank.org:act:CBD65040-30B2-4150-A4D0-7F5BB13EC523.

*Etymology*: The name of the new species refers to its characteristic body shape.

*Description*

[Based on 14 specimens; measurements of the holotype are given in the description; ranges and means for the type-series are provided in Table 2]. Body small, piriform, 346 × 285, with maximum width immediately posterior to ventral sucker; body width/length ratio 0.82. Forebody 181, comprising 52% of body length. Oral sucker 90 × 120, subterminal, round or slightly elongated transversely. Ventral sucker round, equatorial, 82 × 91, smaller than oral sucker; oral/ventral sucker width ratio 1.32. Pseudogonotyl in form of oval ridge of thickened tegument, longitudinally elongate, latero-sinistral to ventral sucker. Prepharynx absent. Pharynx muscular, subspherical, 31 × 27. Oesophagus extremely short (9 in holotype) or indistinct. Intestinal bifurcation at anterior margin of pseudocirrus sac. Caeca short, pre-testicular, posterior ends of caeca usually slightly overlap anterior margins of testes. Testes opposite, subspherical, entire, equatorial, at level of ventral sucker; right testis 71 × 63; left testis 63 × 63. Pseudocirrus sac large, 70 × 71, median, subspherical, between caecal bifurcation and ventral sucker. Posterior part of pseudocirrus sac overlapping with ventral sucker. Pseudocirrus sac contains winding seminal vesicle. Genital atrium small, slightly muscular. Genital pore median, ventral to pseudocirrus sac, at mid-distance between intestinal bifurcation and ventral sucker. Ovary 57 × 50, entire, of irregular shape, dextral to middle axis of body, at level of ventral sucker, partly overlapping with ventral sucker, right testis and pseudocirrus sac. Vitellarium consists of large follicles, distributed in 2 lateral groups between level of posterior margin of oral sucker to middle of testes. Vitelline reservoir small, median, slightly sinistral, overlapped by ventral sucker. Uterus strongly developed, uterine coils almost entirely filling post-testicular space, overlapping posterior margins of testes. Eggs numerous, operculated, 18–19 × 10–11. Excretory pore terminal. Excretory vesicle V-shaped, its arms nearly reaching posterior margins of testes.

**Table 2:** Comparative morphometric data for the 2 new species

Species	*O. piriforme* **n. sp.** (n = 14)			*O. giovannionorei* **n. sp.** (n = 5)		
	Min–max	Mean	CV*	Min–max	Mean	CV*
Body length	296−386	343	6.7	540−609	576	4.3
Body width	241−320	280	8.8	309−393	351	10.5
Body width/body length ratio	0.7−0.9	0.8	7.4	0.54−0.65	0.61	7.9
Forebody length	153−194	176	6.2	240−273	258	4.6
Forebody/body length (%)	47−56	52	5.5	43−48	45	3.8
Oral sucker length	62−93	84	9.4	103−117	108	5.1
Oral sucker width	74−120	103	10.7	103−123	111	7.1
Ventral sucker length	56−90	72	14.2	81−92	85	5.1
Ventral sucker width	59−92	78	13.8	80−91	85	5.0
Oral/ventral sucker width ratio	1.19−1.49	1.35	7.3	1.24−1.43	1.30	6.2
Pharynx length	26−33	29	6.9	29−33	31	7.2
Pharynx width	22−29	26	8.1	28−34	32	7.3
Oesophagus length	0−9	5.6	48.5	−	−	−
Caecal bifurcation to anterior end	83−118	97	12.2	123−146	137	6.9
Pseudocirrus sac length	69−96	79	9.6	74−101	86	11.8
Pseudocirrus sac width	70−93	82	9.5	100−126	113	8.4
Genital pore to anterior end	112−134	124	5.5	170−193	181	4.7
Right testis length	68−87	75	8.3	91−109	102	7.3
Right testis width	52−75	65	10.1	83−101	91	9.1
Left testis length	60−87	68	10.7	84−104	93	9.1
Left testis width	45−72	60	11.7	76−96	88	8.7
Ovary length	57−91	75	12.3	99−114	111	6.0
Ovary width	50−77	66	11.6	90−119	103	13.4
Seminal receptacle length	20−29	24	16.3	27−35	32	9.9
Seminal receptacle width	20−30	24	17.3	27−37	33	11.4
Egg length	18.0−22.0	19.6	5.4	20.0−25.0	21.8	7.0
Egg width	9.0−11.0	9.6	6.2	9.0−12.0	10.3	9.4

Egg measurements were calculated based on 3 eggs from distal part of the uterus of each specimen

*Coefficient of variation

**Remarks**

Based on the combination of morphological features, namely the presence of pseudogonotyl sinistral to the ventral sucker, short caeca, entire testes and median pseudocirrus sac, the characteristics of the new species are consistent with the diagnosis of *Ochoterenatrema.* The measurements of all previously known species were provided by Fernandes et al. (2022), therefore we do not duplicate this information in the present work.

The new species clearly differs from *Ochoterenatrema breckenridgei* (Macy, 1936), *Ochoterenatrema travassosi* (Macy, 1936), *O. gracilis* and *Ochoterenatrema giovannionorei* **n. sp.** (see the description below) in having pre-caecal/caecal vitelline follicles and indistinct oesophagus. The latter 3 species have post-caecal vitelline follicles and a well-defined to very long oesophagus. In addition, the new species differs from *O. breckenridgei* by the ovary situated at the level of ventral sucker (post-acetabular or slightly overlapping ventral sucker in *O. breckenridgei*) and from *O. gracilis* by a dramatically different body shape (compact, piriform in *O. piriforme* **n. sp.** vs elongated, slender in *O. gracilis*). *Ochoterenatrema piriforme* **n. sp.** can be also easily differentiated from *O. giovannionorei* **n. sp.** by the body shape (Fig. 1) and the position of vitelline follicles in relation to ovary (pre-ovarian or slightly overlapping ovary in *O. piriforme* **n. sp.** vs ovarian in *O. giovannionorei* **n. sp.**).

The new species differs from *O. caballeroi* in having much larger sucker length relative to the body length (oral sucker 0.27–0.35, average 0.31 in *O. piriforme* **n. sp.** vs 0.19–0.21, average 0.20 in *O. caballeroi*; ventral sucker 0.19–0.27, average 0.27 in *O. piriforme* **n. sp.** vs 0.12–0.15, average 0.14 in *O. caballeroi*) and having relatively few large vitelline follicles vs numerous small follicles in *Ochoterenatrema caballeroi* Freitas, 1957 (Freitas, 1957).

*Ochoterenatrema piriforme* **n. sp.** differs from *O. diminutum*, *O. labda* and *O. fraternum* in having a distinct piriform body shape vs oval in the latter 3 species, as well as substantially larger suckers (especially the ventral sucker) relative to the body width. The ventral sucker width:body width ratio in the new species is 0.26–0.33, average 0.28 vs 0.13 (original description by Chandler, 1938) to 0.24 (Fernandes et al., 2022) in *O. diminutum*; 0.10 (original description by Caballero, 1943) to 0.25 (Lunaschi, 2002) in *O. labda*; and 0.2 (original description by Freitas & Ibañez, 1963) to 0.24–0.25 (Fernandes et al., 2022) in *O*. *fraternum*. Where the measurements could not be used from the text or tables in the publications, we measured the illustrations. Lower sucker width to body ratios in some of the original descriptions likely reflect the effect of specimen flattening under cover slip which was often used in the past.

Lastly, the new species differs from *O. sphaerula* in having a piriform body vs nearly spherical body in *O. sphaerula* and ovary situated at the level of ventral sucker compared to between ventral and oral suckers in *O. sphaerula*. In addition, the caeca in *O. piriforme* **n. sp.** terminate at the level or below the level of the anterior margin of ventral sucker, while in *O. sphaerula* they end at a significant distance anterior to the ventral sucker (Fernandes et al., 2022).

The DNA sequences demonstrate differences between *O. piriforme* **n. sp.** and 5 other species of *Ochoterenatrema* sequenced so far (Table 3). In the 1,116 bp long alignment of the partial 28S gene sequences, *O. piriforme* **n. sp.** differs from *O.* cf. *labda*, *O. gracilis*, *O. diminutum* and *O.* *giovannionorei* **n. sp.** by 0.3–1% of nucleotide positions and is most distant from *O. diminutum*. At the same time, the 28S sequence of the new species does not have differences from *O. fraternum* in the sequenced fragment of 28S (Table 3). The divergence levels in the partial sequences of the *cox*1 gene are much greater. *Ochoterenatrema piriforme* **n. sp.** differs from other species by 8.7–19.8% being closest to *O. fraternum* from Ecuador and most distant from *O*. cf. *labda* from Brazilian Pantanal (Table 3).

**Table 3:** Divergence percentages resulting from pairwise sequence comparisons of 395 base pair long alignment of the partial *cox*1 gene (above diagonal) and 1,116 base pair long alignment of the partial 28S gene (below diagonal)

	1. OM641990	2. OM641988	3. OM641989	4. OM641991	5. PP069737	6. PP069738
1.* O.* cf. *labda* OM574913	–	18.5	20.6	16.8	19.8	18.0
2. *O. gracilis* OM574910	1.0	–	14.2	9.7	10.9	13.0
3. *O. diminutum* OM574911	1.2	1.0	–	14.5	13.5	13
4. *O. fraternum* OM574914	0.9	0.4	1.0	–	8.9	7.9
5. *O. piriforme* **n. sp.** PP069556	0.9	0.4	1.0	0	–	8.7
6. *O. giovannionorei* **n. sp.** PP069557	1.1	0.5	1.2	0.3	0.3	–

GenBank numbers for *cox*1 sequences are provided in the top row, GenBank numbers for 28S sequences are provided in the first column

***Ochoterenatrema giovannionorei*** **n. sp.**

(Fig. 1D–F)

*Type-host*: *Molossus molossus* (Pallas) (Chiroptera, Molossidae Geoffroy).

*Type-locality*: Yasuní National Park, Orellana Province, Ecuador (0° 40′ 27.8″S; 76° 23′ 50.32″W).

*Type-material*: The type series consists of 5 fully mature specimens. Holotype: QCAZI 278991, small intestine, Estación Científica Yasuní, Yasuní , Orellana Province, Ecuador, 12 March 2016, coll. J. Cook; paratypes: QCAZI 278992-278995, labels identical to the label of holotype.

*Site in host*: Small intestine.

*Representative DNA sequences in GenBank*: PP534962 (28S), PP534965 (*cox*1).

*ZooBank registration:* urn:lsid:zoobank.org:act:36AD7C8D-5CB3-4785-95D8-58134D2FFE0F.

*Etymology*: The species is named after Dr. Giovanni Onore in recognition of his contributions to the conservation of nature in Ecuador and for being a mentor of 1 of the authors, Carlos Carrión-Bonilla.

*Description*

[Based on 5 specimens; measurements of the holotype are given in the description; ranges and means for the type-series are provided in Table 2]. Body small, oval, narrowing at both ends, 540 × 309, with maximum width at level of ventral sucker; body width/length ratio 0.57. Forebody 240, comprising 44% of body length. Oral sucker subterminal, round, 107 × 103. Ventral sucker round, equatorial, 85 × 82, smaller than oral sucker; oral/ventral sucker width ratio 1.26. Pseudogonotyl in form of oval ridge of thickened tegument, elongate, sinistral to ventral sucker. Prepharynx absent. Pharynx muscular, spherical, 33 × 32. Oesophagus indistinct. Intestinal bifurcation anterior to pseudocirrus sac. Caeca short, pre-testicular, posterior ends of caeca reach anterior margins of testes. Testes opposite, subspherical, entire, just anterior to middle of body; right testis 91 × 83; left testis 87 × 76. Pseudocirrus sac 74 × 109, median, slightly transversely elongated, between ventral sucker and caecal bifurcation subspherical, between caecal bifurcation and ventral sucker. Pseudocirrus sac containing winding seminal vesicle. Genital pore median, ventral to pseudocirrus sac. Ovary 114 × 117, entire, irregularly shaped, median, partly overlapping both ventral sucker and pseudocirrus sac. Seminal receptacle small, spherical, 27 × 27; Laurer’s canal and Mehlis` gland not observed. Vitellarium consists of large follicles, distributed in 2 post-caecal lateral groups at level of testes, overlapping testes. Uterus strongly developed, uterine coils almost entirely filling post-testicular space, slightly overlapping posterior margins of testes. Eggs numerous, operculated, 20–21 × 9–10. Excretory pore terminal. Excretory vesicle V-shaped, its arms reaching posterior margins of testes.

**Remarks**

Based on the combination of morphological features, namely the presence of pseudogonotyl sinistral to the ventral sucker, short caeca, entire testes and median pseudocirrus sac, the characteristics of the new species are consistent with the diagnosis of *Ochoterenatrema.*

The new species can be readily differentiated from *O. diminutum*, *O. labda*, *O. fraternum*, *O. caballeroi*, *O. sphaerula* and *O. piriforme* **n. sp.** by post-caecal vitelline follicles which overlap the testes while in all of these species the vitelline follicles are completely or partially pre-caecal, not overlapping or only slightly overlapping testes. Of these 6 species, *O. giovannionorei* **n. sp.** is morphologically (and genetically, see below) closest to *O. fraternum.* In addition to the clear difference in the position of vitelline follicles, the 2 species also differ in the position of ovary. In *O. giovannionorei* **n. sp.** the ovary is median, overlapping with ventral sucker, while in *O. fraternum* the ovary is lateral to the ventral sucker.

*Ochoterenatrema giovannionorei* **n. sp.** differs from *O. gracilis* in having a very different body shape (much more elongated, slender in *O. gracilis*), vitelline follicles at level of testes (post-testicular in *O. gracilis*) and indistinct oesophagus (very long in *O. gracilis*).

The new species differs from both *O. breckenridgei* and *O. travassosi* by lacking a distinct oesophagus (well-defined or long oesophagus in *O. breckenridgei* and *O. travassosi*). The oral sucker in the new species is significantly larger than the ventral sucker while in *O. breckenridgei* the suckers are nearly equal (oral to ventral sucker width ratio in *O. giovannionorei* **n. sp.** is 1.24–1.43, average 1.30 vs 1.0 (original description by Macy, 1936) to 1.1 (re-description by Lotz & Font, 1983). The ovary in the new species is dorsal to the ventral sucker while *O. breckenridgei* has a post-acetabular ovary. Caeca reach testes in the new species, but not in *O*. *travassosi*.

The DNA sequences show significant differences between *O. giovannionorei* **n. sp.** and 5 other species of *Ochoterenatrema* sequenced so far (Table 3). In the 1,116 bp long alignment of the partial 28S gene sequences, *O*. *giovannionorei* **n. sp.** differs from all other sequenced species by 0.3–1.2% of nucleotide positions and is most distant from *O. diminutum* (Table 3). The divergence levels in the partial sequences of the *cox*1 gene are much greater. *Ochoterenatrema giovannionorei* **n. sp.** differs from other species by 7.9–18.0%, being closest to *O.* *fraternum* from Ecuador and most distant from *O*. cf. *labda* from the Brazilian Pantanal (Table 3).

*Phylogenetic analysis*

**Fig. 2:**
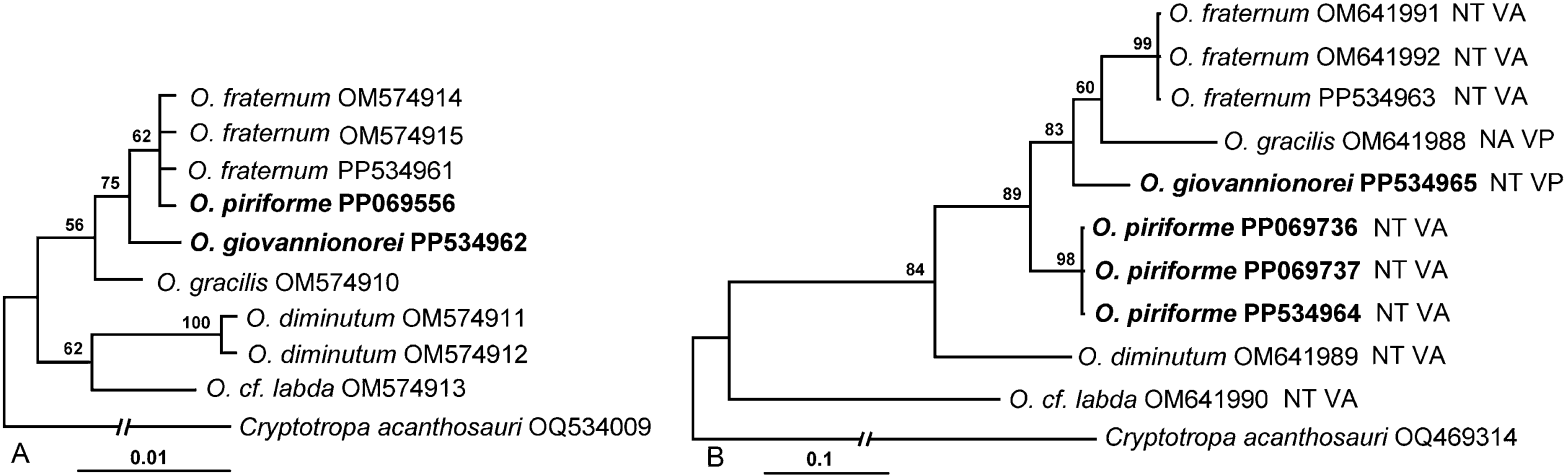
Phylogenetic relationships among *Ochoterenatrema* spp. resulting from Bayesian analysis of partial sequences of 28S gene (A) and *cox*1 gene (B). Species sequenced in the present paper are in bold. Branch length scale bar indicates the number of substitutions per site. NA – Nearctic, NT – Neotropics, VA – pre-testicular vitelline follicles; VP – testicular/post-testicular vitelline follicles.

The alignments of partial 28S sequences used in the phylogenetic analysis included sequences of 6 *Ochoterenatrema* spp. and *C. acanthosauri* (Table 1). The final alignment was 1,165 bp long with 51 positions excluded from the analysis due to ambiguous homology. The BI analysis resulted in an overall weakly resolved tree in which the basal polytomy included branches of *O*. cf. *labda*, *O. diminutum*, *O. gracilis* and weakly supported (75%) clade of *O*. *giovannionorei* **n. sp.** + (*O. fraternum* + *O*. *piriforme* **n. sp.**) (Fig. 2A). The internal topology of the latter clade was completely resolved due to the lack of interspecific variability in the 28S gene between *O. fraternum* + *O*. *piriforme* n. sp.

The alignments of partial *cox*1 sequences used in the phylogenetic analysis included 10 sequences of 6 *Ochoterenatrema* spp. and *C. acanthosauri* as the outgroup (Table 3). The final alignment was 395 bp long; no exclusion set was necessary. The BI analysis resulted in a relatively well-resolved tree in which *O*. cf. *labda* appeared as a sister group to the somewhat weakly supported (84%) clade containing all remaining species (Fig. 2B). Most topologies within the latter clade were well supported with all species well separated from each other. In this clade, *O. diminutum* appeared as the sister taxon to the cluster containing *O. piriforme* **n. sp.** and a 83% supported polytomy containing *O. giovannionorei* **n. sp.**, *O. gracilis* and *O. fraternum*.

## Discussion

There has been a recent increase in studies of bat helminths in the Neotropics, in some contrast to the lack of recent similar studies in the Nearctic (Achatz et al., 2018, 2020; Tkach et al., 2018, 2019; Fernandes et al. 2019, 2021a,b, 2022; Panti-May et al., 2021; Moguel-Chin et al., 2023, 2024; Kinsella et al., 2024). All these studies have invariably demonstrated that the helminth fauna of bats in the Western Hemisphere remains understudied and that these hosts likely harbor a much richer fauna of helminth parasites than currently known. *Ochoterenatrema* is 1 of the most broadly distributed genera of bat digeneans in the Western Hemisphere. Fernandes et al. (2022) posited that, based on the high diversity of bats in the Western Hemisphere, especially the Neotropics, the diversity of *Ochoterenatrema* was far from being exhausted and the number of known species will likely increase if greater diversity of bats from a broader geographic range are examined for helminths. In the present study, we have further expanded our knowledge on diversity, geographic distribution, and host ranges of lecithodendriid digeneans belonging to the genus *Ochoterenatrema*.

The 2 new species described herein are characterized by unique morphological features including the compact, pear-shaped morphotype in *O. piriforme* **n. sp.**, which has not previously been reported in this genus. As the result, the genus now contains digeneans demonstrating a rather extreme range of body shapes, from nearly spherical (*O. sphaerula*) to pear-shaped (*O. piriforme* **n. sp.**), several variations of oval (*O. breckenridgei*, *O. caballeroi*, *O. diminutum*, *O. fraternum*, *O. giovannionorei* **n. sp.**, *O. labda*), and elongated slender form (*O. gracilis*). Likewise, the position of some internal organs varies quite dramatically. For instance, the vitelline follicles are pre-caecal/caecal in the majority of species (*O. labda*, *O. diminutum*, *O. fraternum*, *O. caballeroi*, *O. sphaerula*, *O. piriforme* **n. sp.**), while the remaining species (*O. breckenridgei*,* O. giovannionorei* **n. sp.**, *O. gracilis*) have post-caecal vitelline follicles.

The ovary also can be found in different positions in relation to the ventral sucker, from being overlapped by the ventral sucker to situated lateral, anterior, or posterior to it. In addition, the length of the oesophagus is highly variable among different *Ochoterenatrema* species. Only the short caeca, testes at level of ventral sucker and the presence of the pseudogonotyl are stable characters across the genus, the latter being the only unique character readily differentiating *Ochoterenatrema* spp. from other related genera.

Among lecithodendriids, as well as other xiphidiatan digeneans, the wide range of morphological differences often results in separating species in different genera. However, currently available evidence suggests that *Ochoterenatrema* is clearly monophyletic. This leads to a suggestion that the interrelationships and systematic position of many species in several lecithodendriid genera, especially *Lecithodendrium* and *Paralecithodendrium* (and its present synonyms, whether properly justified or not) need to be re-evaluated using DNA sequence data and morphology based on newly collected quality specimens. Many members of these genera seem to differ only in the pre- or post-testicular position of vitelline follicles, although some species, or even variants within a species, show a variation from lightly pre-testicular to testicular and post-testicular position, e.g., in *Paralecithodendrium hurkovaae* (Dubois, 1960) (see Tkach et al., 2003).

The discovery of *O. giovannionorei* **n. sp.** in South America addresses 1 of the observations of Fernandes et al. (2022), who noted that at the time of their publication the species with post-testicular vitelline follicles or vitelline follicles overlapping testes were known only from North America. They also wrote that it would be interesting to test the monophyly of species with this type of vitelline follicle arrangement. *Ochoterenatrema giovannionorei* **n. sp.** has vitelline follicles positioned at the level of testes and is found in South America (Ecuador). Most specimens of *O. piriforme* **n. sp.** have clearly pre-testicular follicles only slightly overlapping testes; therefore we consider their position pre-testicular in this species. Although *O*. *gracilis* and *O*. *giovannionorei* **n. sp.** appear in the same clade (Fig. 2B), they do not form a monophyletic group because *O. fraternum*, a species with pre-testicular vitellarium, is 1 of the derived taxa in this clade.

The pairwise nucleotide comparisons among newly obtained and previously published 28S and *cox*1 sequences of *Ochoterenatrema* spp. available for our analysis have demonstrated that in this genus, similar to some other previously studied digenean taxa (e.g., Gordy et al., 2017; Hernández-Mena et al., 2017; Achatz et al., 2022, 2023b), 28S gene is not always variable enough to reliably differentiate between congeneric species (Table 3). Partial 28S sequences of *O. fraternum*, *O*. *giovannionorei* **n. sp.** and *O*. *piriforme* **n. sp.** were identical, while the overall levels of divergence between 6 species in our analysis did not exceed 1.2% (between *O. diminutum* and *O*. cf *labda*). In contrast, as previously shown in multiple digenean taxa, *cox*1 sequences proved to be much more suitable for differentiation between congeneric species (e.g., Gordy et al., 2017; Locke et al., 2018; Achatz et al., 2022, 2023a, b), in this case *Ochoterenatrema* spp. (Table 3). The interspecific divergence in our dataset varied from 3.3% between *O. fraternum* and *O*. *giovannionorei* **n. sp.** to 20.5% between *O. diminutum* and *O*. cf *labda*. All other species in our dataset were more distant from *O*. cf. *labda* than from any other species. *Ochoterenatrema* cf. *labda* was collected in the Brazilian Pantanal (Fernandes et al., 2022), while most of the remaining species were collected in Ecuador with the exception of *O*. *gracilis* which originated from Oklahoma, United States (Nearctic). Despite being on the same continent, the geographic and environmental isolation between the Humid Tropical Rainforest of Ecuador and the Pantanal seems to have greater effect on genetic/phylogenetic divergence between *Ochoterenatrema* spp. than between Ecuador and southern United States.

Unfortunately, only a single *Ochoterenatrema* species from Nearctic, *O. gracilis*, has been sequenced so far (Fig. 2B). In the *cox*1 tree, *O. gracilis* was nested among several Neotropical species (Fig. 2B). Insufficient representation of *Ochoterenatrema* from Nearctic and Neotropical bat species and across more localities prevents us from making broader biogeographic considerations with regard to the levels of genetic divergence and phylogenetic affinities among species from the Nearctic and Neotropical biomes.

Parasitological surveys of more bat species across wider geography will provide critical material to stimulate sequencing of other *Ochoterenatrema* species from the Nearctic (e.g., *O. breckenridgei*, *O. travassosi*) to more rigorously address systematic and biogeographic questions.

Two morphologically easily distinguishable species, *O. fraternum* and *O*. *giovannionorei* **n. sp.** differed by only 3.3% of nucleotide positions in the sequenced *cox*1 region. This provides evidence that even a divergence as low as 3.3% in *cox*1 provides a sufficient basis to differentiate between species in this digenean lineage. No intraspecific variability was detected between 2 *cox*1 sequences of *O. piriforme* obtained in the present study, similar to what was reported by Fernandes et al. (2022), who also did not find intraspecific variability in *cox*1 between 5 sequenced *O. fraternum*.

The resolution of the tree topology in our 28S analysis was low and resulted mostly in polytomies. This can be readily explained by the low variability of that region. Although the *cox*1 tree was much better resolved, it was less supported than could be expected from this gene at this taxonomic level. We believe that using a longer part of the *cox*1 gene and additional independent markers will allow the assessment of the interrelationships between *Ochoterenatrema* spp. more confidently. We were limited to this fragment because we were not able to obtain longer sequences from some of the species despite multiple attempts. The use of next-generation sequencing technologies should allow future studies to overcome difficulties in obtaining longer sequences and generate data for multi-gene phylogenies. Based on the rapid increase of the number of known *Ochoterenatrema* species despite still limited geographic and host coverage, we believe that a significant part of *Ochoterenatrema* diversity still awaits discovery.

## Data Availability

Specimens used in this work have been deposited to publicly accessible museum collections and novel sequence data have been deposited in GenBank.
